# Thermodynamic Behaviors of Adsorbed Methane Storage Systems Based on Nanoporous Carbon Adsorbents Prepared from Coconut Shells

**DOI:** 10.3390/nano10112243

**Published:** 2020-11-12

**Authors:** Ilya E. Men’shchikov, Andrey V. Shkolin, Evgeny M. Strizhenov, Elena V. Khozina, Sergey S. Chugaev, Andrey A. Shiryaev, Anatoly A. Fomkin, Anatoly A. Zherdev

**Affiliations:** 1 Research Institute of Power Engineering, Bauman Moscow State Technical University, Baumanskaya 2-ya str. 5, 105005 Moscow, Russia; shkolin@bk.ru (A.V.S.); strizhenov@list.ru (E.M.S.); chugaev@bmstu.ru (S.S.C.); azherdev@bmstu.ru (A.A.Z.); 2 Frumkin Institute of Physical Chemistry and Electrochemistry, Russian Academy of Sciences, Leninskii Prospect, 31, build. 4, 119071 Moscow, Russia; khozinaelena@gmail.com (E.V.K.); a_shiryaev@mail.ru (A.A.S.); fomkinaa@mail.ru (A.A.F.)

**Keywords:** adsorption, nanoporous carbon adsorbents, methane storage, thermodynamic of adsorption

## Abstract

The present work focused on the experimental study of the performance of a scaled system of adsorbed natural gas (ANG) storage and transportation based on carbon adsorbents. For this purpose, three different samples of activated carbons (AC) were prepared by varying the size of coconut shell char granules and steam activation conditions. The parameters of their porous structure, morphology, and chemical composition were determined from the nitrogen adsorption at 77 K, X-ray diffraction (XRD), small-angle X-ray scattering (SAXS), and scanning electron microscopy (SEM) measurements. The methane adsorption data measured within the temperature range from 178 to 360 K and at pressures up to 25 MPa enabled us to identify the most efficient adsorbent among the studied materials: AC-90S. The differential heats of methane adsorption on AC-90S were determined in order to simulate the gas charge/discharge processes in the ANG system using a mathematical model with consideration for thermal effects. The results of simulating the charge/discharge processes under two different conditions of heat exchange are consistent with the experimentally determined temperature distribution over a scaled ANG storage tank filled with the compacted AC-90S adsorbent and equipped with temperature sensors and heat-exchanger devices. The amounts of methane delivered from the ANG storage system employing AC-90S as an adsorbent differ from the model predictions by 4–6%. Both the experiments and mathematical modeling showed that the thermal regulation of the ANG storage tank ensured the higher rates of charge/discharge processes compared to the thermal insulation.

## 1. Introduction

The advantages of natural gas (NG) as an attractive fuel alternative include its availability, low cost and potential for reducing greenhouse gas emissions. However, the NG energy density (energy per volume) is 0.12% of that of gasoline [1]. Therefore, NG stored as transportation fuel at 250 bar (nominal working pressure) requires 3.5 times greater storage volume to yield the same energy as a liter of gasoline [2]. Therefore, the large-scale application of an NG vehicle depends on the NG storage method and volumetric capacity of an on-board fuel tank. The conventional methods of NG storage in liquified (LNG) and compressed (CNG) states have some disadvantages, such as the high fire and explosive hazards. CNG needs a high storage pressure from 200 to 300 bar, and LNG storage at 112K requires special equipment [1,2,3,4,5]. Adsorbed natural gas (ANG) storage is one of the strategies to increase the volumetric capacity of the NG vehicle by binding methane molecules to an adsorbent through a weak van der Waals interaction (physical adsorption) at relatively low pressures [1,2,3,4,5]. It should be noted that adsorption phenomena form the basis of many industrial processes. For example, pressure swing adsorption is used in the biogas upgrading process and allows the separation of methane from nitrogen, oxygen, and carbon dioxide resulting in a methane purity of 97.13% [6].

Adsorbents used in advanced systems for ANG storage must meet high requirements on specific adsorption capacity, cyclic stability, energy efficiency, and safety [1,3,4,5]. Conventional adsorption techniques of refining, separation, and drying of gases and liquids use only 15–30% of the total adsorption capacity of adsorbents. In contrast, ANG systems utilize 100% of the adsorption potential of a microporous adsorbent, which is determined by its specific micropore volume, pore size distribution, surface chemistry, and adsorption energy [3,4,5]. The systems, imposing such challenging working conditions for adsorbents, should be assigned to a separate class of high-energy adsorption systems (HEAS). High performance of an adsorbent intended for HEAS requires a “tailored” porous structure [3,4,5,7,8,9]. In terms of the Dubinin theory of volume filling of micropores (TVFM) [10], this means that the efficient adsorbent must possess an optimal micropore half-width or radius *x*_0_, high micropore volume (*W*_0_), and characteristic energy of adsorption (*E*_0_) [11,12]. Microporous activated carbons (AC) fit into this group of adsorbents due to their chemical, mechanical, and thermal stability and the possibility of tuning their structural and energy characteristics by various activation methods [7,8,13,14,15,16,17]. In addition to activation methods, the performance of carbon adsorbent in HEAS applications depends greatly on the type of a precursor, its morphology, and chemical composition [3,18,19,20]. A number of studies have shown that the use of biomass waste such as coconut shell made it possible to produce efficient low ash carbon adsorbents for HEAS, in particular ANG systems, with high values of micropore volume and packing density [17,21,22,23]. The scale of their utilization is determined by the abundance of raw materials and a high level of technological readiness for industrial production.

In addition to the textural and adsorption properties of adsorbent, the thermal effects of adsorption/desorption are critical factors for ANG storage performance during the charge/discharge processes [24]. Indeed, the adsorption/desorption processes are accompanied by the heat release/absorption effects, which change the temperature of the adsorbent. At high charge rates, the temperature inside the ANG tank can rise by 80 °C slowing down the methane uptake. Conversely, at high discharge rates, the temperature drop is 40 °C, and, hence, the efficiency of the ANG system decreases by 25% compared to that under isothermal conditions [25]. Mathematical modeling becomes a widely used tool for studying various processes in ANG systems. Different models [26,27,28,29] were used for analyzing the thermal effects observed experimentally upon the charge/discharge processes in ANG systems comprising adsorbents with different properties, ANG tank configuration, and operational conditions [14,30,31,32]. Recently, the authors developed a mathematical model of the circuit charging process of a flow-type ANG storage system [33]. Therefore, a practical engineering solution for the ANG facilities must consider the thermal effects of adsorption/desorption [34,35,36,37,38,39]. In this context, thermodynamic functions of adsorption systems, operating under conditions of high pore filling with adsorbate at high pressures, i.e., the HEAS conditions are of particular interest [40,41]. In this case, the isosteric heat of adsorption is an essential parameter, the correct evaluation of which defines the success of using particular mathematical models for calculating the characteristics of a scaled ANG system [42,43].

Thus, the aim of the present study is to find optimal conditions ensuring high performance of the ANG system employing activated carbon as an adsorbent. For this purpose, we carried out a comprehensive investigation of the ANG system based on nanoporous activated carbons prepared from coconut shells. We started from the synthesis of the adsorbents, characterized their textural properties, and measured methane adsorption within a wide *P,T*-range. Finally, we performed the theoretical and experimental examinations of the thermodynamic behaviors of a scaled ANG system operating under various conditions of heat exchange relevant for applications.

## 2. Materials and Methods

### 2.1. Adsorbent

The nanoporous AC adsorbents were prepared from coconut shells (CNS) in two stages, including carbonization of the crushed precursor at about 873 K (1), and steam activation of char at 1123–1273 K (2). Three different samples were obtained by changing the activation time from 60 (AC-60L) to 90 min (AC-90S and AC-90L) and via the size fractionation into relatively small (0.7–1.1 mm for AC-90S) and large (0.9–2.4 mm for AC-90L) granules. The sizes of AC-60L granules were between 1.9 and 3.0 mm. The sample designation indicates the activation time: 60 or 90 min, and granule size fraction: small (S) or large (L) size. The variations in the synthesis conditions led to the differences in the burn-off degree (Ω, wt%, calculated as percent weight loss upon activation) and, consequently, in the porous structure of the adsorbents.

The packing density (*d*, kg/L) of the prepared adsorbents was evaluated as the ratio of the mass of the sample and the volume occupied by the sample in a measuring cylinder according to the procedure of measuring weight and dimensions described in ISO 60 and ISO 697 standards [44,45].

In order to increase the packing density of adsorbent in the ANG system, the AC-90S granules were compacted with a polymer binder into hexagonal monolithic prisms. The content of the binder in the prisms was about 8 wt%. The shaping method was described in detail in [37]. It was shown that this procedure does not lead to a noticeable reduction of the adsorption capacity of activated carbons. The density of the AC-90S prism was 730 kg/m^3^; the average packing density of the adsorbent in the ANG tank was about 650 kg/m^3^.

### 2.2. Adsorptive

The adsorptive gas used in the experiments was high purity (99.999%) methane. Methane has the following physicochemical properties: molecular mass *M* = 16.0426 g/mol; boiling temperature *T*_0_ = 111.66 K; critical temperature *T*_cr_ = 190.77 K; critical pressure *P*_cr_ = 4.641 MPa [46].

Natural gas used in internal-combustion engines with technical specifications determined by the Russian State Standards (GOST 27577-2000) was used to investigate the heat effects of methane charge/discharge processes by a specially designed ANG test bench. It contained ~96% methane; ~2% nitrogen; the total content of hydrocarbons higher than C_2+_ was about 2%; the amounts of water and CO_2_ were less than 100 ppm and had no significant impact on the operation of the ANG test bench.

### 2.3. Methods

#### Structural Characterization of Coconut Shell (CNS)-Derived Carbon Adsorbents

The porous structure parameters of the adsorbents were evaluated from the standard nitrogen adsorption data at 77 K measured using a Quantachrome Autosorb iQ multifunctional surface area analyzer. The structural and energy characteristics (*W*_0_, *E*_0_, and *x*_0_) of the samples were calculated from the nitrogen adsorption data using the Dubinin–Radushkevich (D–R) Equation [10]. The Brunauer–Emmet–Teller (BET) [47] and Kiselev [48] equations were used to calculate the specific surface area *S*_BET_ and volume of mesopores *W*_meso_. The pore size distribution in the adsorbents was derived using the non-local density functional theory (NLDFT) for a combined slit + cylindrical pore model [49].

Surface morphology and chemical composition of mechanically crushed samples were studied by scanning electron microscopy (SEM) using a Quanta 650 FEG (FEI, Company, Hillsboro, OR, USA) equipped with an Oxford Inca energy-dispersive X-ray (EDX) system for chemical analysis operating at 30 kV accelerating voltage.

The phase composition of the samples was analyzed using the X-ray diffraction (XRD) patterns collected by an Empyrean (Panalytical BV, Almelo, The Netherlands) diffractometer in Bragg–Brentano geometry using Ni-filtered CuKα-radiation (λ_Cu_ = 0.1542 nm) in the 2θ angular range from 10 to 120°. The samples were ground to powder; no binder was employed. The International Centre for Diffraction Data PDF-2 (ICDD PDF2) database was used for phase identification. Small-angle X-ray scattering (SAXS) was recorded using a dedicated SAXSess diffractometer (Anton Paar, Graz, Austria). The measurements were performed under vacuum in transmission geometry; scattering vectors (q = 4sinπ(Θ)/λ) were measured from 0.1 to 27 nm^−1^; the scattering patterns were recorded using an image plate desmeared using standard procedures.

Three original adsorption setups designed in Frumkin Institute of Physical Chemistry and Electrochemistry, Russian Academy of Science were used to measure methane adsorption equilibria onto the carbon adsorbents within the pressure range from 5 to 25 MPa and at the temperatures from 178 to 360 K by the volumetric-gravimetric method [50,51,52]:

1. semi-automatic adsorption weight vacuum unit (from 5 Pa to 0.1 MPa, gravimetric method; the accuracy of ±1.5% with a confidence level of 0.95) [50];
2. universal adsorption-dilatometer setup (0.1–6 MPa, volumetric method, the accuracy of ±3% with a confidence level of 0.95) [51];
3. original volumetric-gravimetric high-pressure setup (0.2–25 MPa, the accuracy ±5% with a confidence level of 0.95) [52].

Before the experiments, the adsorbents were regenerated at the temperature of 673 K and pressures less than 1 kPa. A detailed description of the volumetric and gravimetric adsorption experiments can be found in [50,51,52]. The value of methane adsorption was determined as an amount of gas adsorbed from a measuring unit corrected to a skeletal volume of the adsorbent evaluated from the helium pycnometry experiments [53] and a micropore volume calculated from the nitrogen adsorption data at 77 K by the D–R equation. The fraction of meso- and macropores was insignificant, so their contribution to the total adsorption is negligible.

Thus, the resultant value is an absolute (total content) methane adsorption determined, as follows:

*a* = (*N* − (*V* − *V*_a_) × *ρ*_g_)/(μ × *m*_0_). (1)

Here, *N* is the amount of methane injected into a measuring unit, [g]; *V* is the total geometric volume of the measuring system, [cm^3^]; *V*_a_ is the volume of an adsorbent with micropores, [cm^3^]; *ρ*_g_ is the density of gaseous phase, [g/cm^3^] at specified values of pressure *P* and temperature *T*; μ is the molar mass of gas, [g/mmol]; *m*_0_ is the mass of a regenerated adsorbent, [g]. The volume of the adsorbent with micropores *V*_a_, [cm^3^], was calculated as a sum of a volume determined via helium pycnometery, *V*_He_, [cm^3^], and product *m*_0_ × *W*_0_, where micropore volume *W*_0,_ [cm^3^/g], is evaluated from the data on nitrogen adsorption by the D–R equation.

Prior to a series of experimental studies, we assessed the repeatability of methane adsorption data for all three setups by conducting two measurements of methane adsorption on the AC-90S samples on each setup. The repeatability of measurements amounted to 0.6%, 2.1%, and 3.4% for the aforementioned (1), (2), and (3) setups, respectively.

### 2.4. Experimental Test Bench and Approach to Study the Heat Effects of Methane Charge/Discharge Processes in the Adsorbed Natural Gas (ANG) System

**Figure 1:**
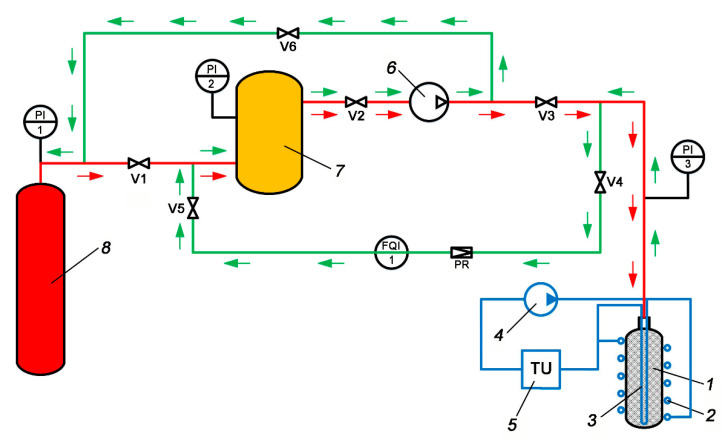
Scheme of the test bench for investigation of the thermal effects of the methane charge/discharge processes of the adsorbed natural gas (ANG) system. 1—storage tank (adsorber) filled with a shaped activated carbon (AC), 2—external heat-exchanger, 3—internal heat-exchanger, 4—coolant circuiting pump, 5—thermostatic unit TU, 6—gas compressor, 7—low-pressure receiver, 8—high-pressure receiver, V1—V6—gas valves, PR—pressure regulator, PI1—3–gauges, FOI1—gas meter. The red arrows indicate the gas charge process; the green arrows correspond to the gas delivery process.

An experimental test bench (Figure 1) was developed to study the influence of thermal effects on the performance of the ANG system during the charge/discharge processes.

**Figure 2:**
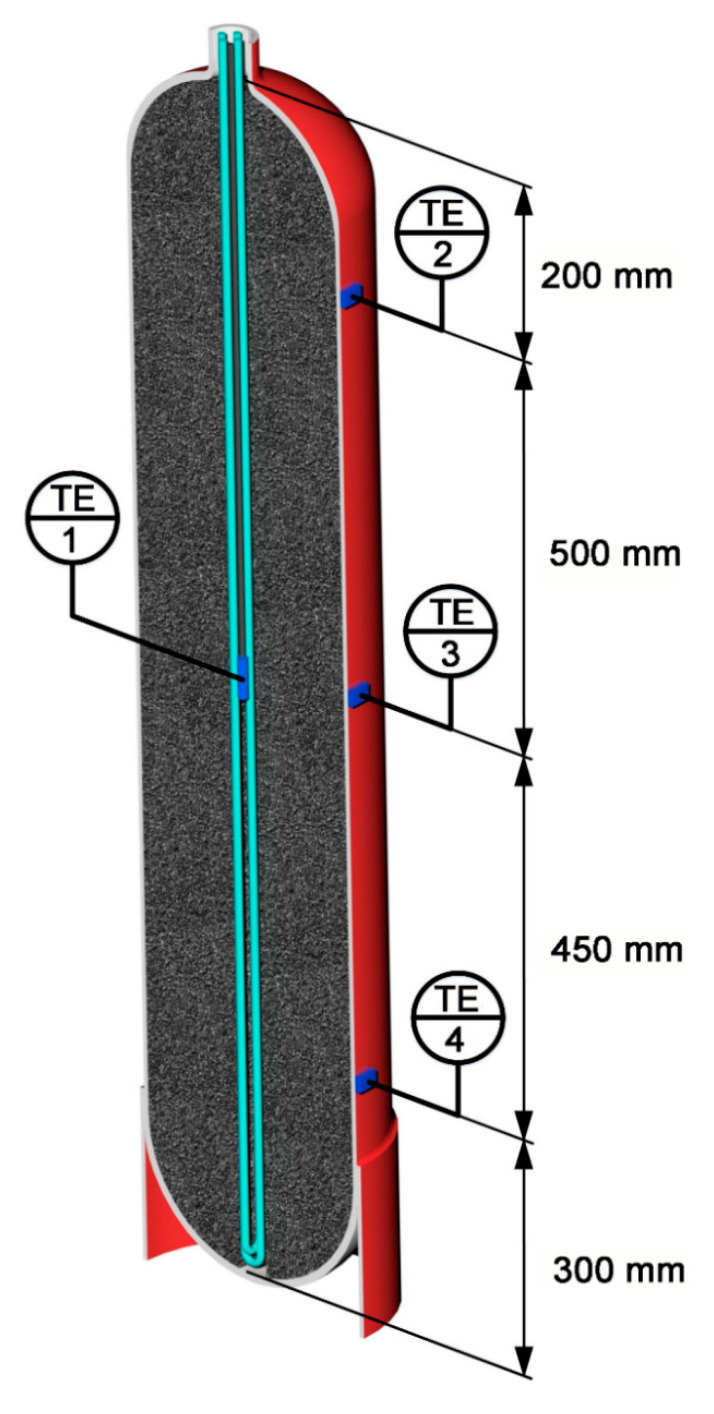
The longitudinal section of the ANG tank packed with carbon adsorbent and equipped with Table 1. The external heat-exchanger is not shown. The sensors are isolated from the heat exchangers and are located between the coil turns.

The main element of the scheme was an ANG storage tank or adsorber (1) with a volume of about 98 L equipped with an external (2) and internal (3) heat-exchangers with a circulating coolant (96% aqueous ethanol solution). The adsorber had a cylindrical shape with an outer diameter and surface area of 305 mm and 1.4 m^2^, respectively; its total external surface area, including bearing surface, was 1.8 m^2^. The external heat exchanger (2) was a 26 m-long tube with the 1/2″ (12.7 mm) outer and 11.08 mm inner diameters and an inner surface area of 0.905 m^2^ that encircled the adsorber. The internal heat-exchanger (3) consisted of two U-tubes with an outer/inside diameter of 9/7 mm and a total length of 5.92 m. The outer surface area of the internal heat-exchanger was 0.167 m^2^, its inner surface–0.130 m^2^. The use of the external and internal heat exchangers made it possible to reduce temperature gradients inside the adsorber upon forced cooling and heating. Circulation of the coolant was driven by a pump (4). The temperature of the ethanol solution was maintained by a thermostatic unit TU (5) within the range from 20 to 23 °C. The adsorber was thermally insulated with an elastomeric insulating material layer to reduce internal temperature gradients. The internal heat exchanger was placed into a cylindrical hollow space with a diameter of 40 mm coaxial with the adsorber (see Figure 2). This hollow space reduced the useful (available for adsorbent) volume by 1.6%. The adsorber was packed with AC-90S in the form of a hexagonal monolithic prism most densely. The ANG tank was refueled using the gas compressor (6). The low-pressure receiver (7) ensured the required pressure at the compressor suction. The high-pressure receiver (8) was used as a gas collector in the experiments. Methane pressure in the low- and high-pressure receivers was monitored using the gauges PI1–3.

Prior to the experiments, the ANG tank was regenerated by desorptive displacement and thermal vacuum desorption. All elements of the test bench were also degassed.

For the experiments, the ANG tank was refueled with natural gas up the pressure of 10–11 MPa for 1.5–2 h. The discharge cycle was carried out at the pressure drop from 10 to 0.15 MPa during 2 h. The pressure was reduced to a required level by a pressure regulator (PR), and natural gas was pumped to the high-pressure receiver by the compressor. The amount of delivered gas was monitored using a gas meter FQI1. The charge/discharge procedures were performed using a compressor, providing stable gas consumption. Gas refueling and delivery were conducted stepwise, employing at least two stages, the first was examined in more detail. At the first stage, the pressure rose and dropped to a certain level, after which the adsorber was closed and cooled/heated to a certain temperature. Then, additional portions of gas were fed to/withdrawn from the adsorber to provide the complete gas charge/discharge cycle.

The ambient temperature varied from 19 to 30 °C.

Resistive temperature sensors were used to control the temperature inside (TE1 in the central hollow space) and on the external surface of the adsorber (TE2–4) at different heights (see Figure 2). During the experiments, the temperature inside the adsorber varied from −10 °C to +60 °C.

## 3. Results and Discussion

### 3.1. Porous Structure of CNS-Derived Carbon Adsorbents

**Figure 3:**
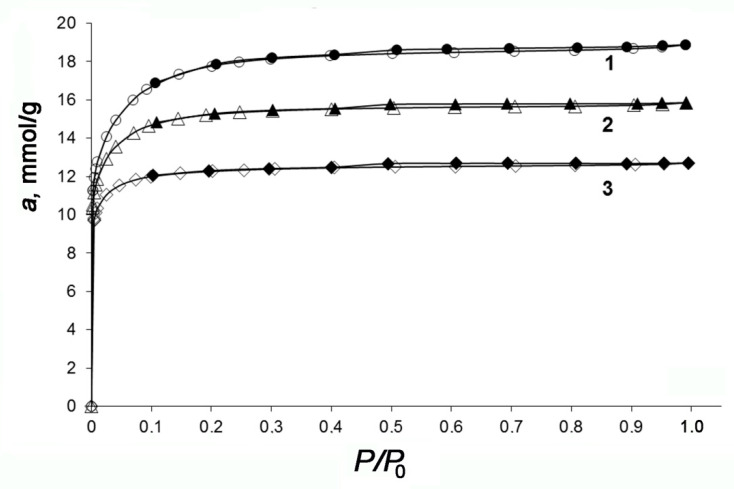
Nitrogen adsorption (dark symbols)/desorption (light symbols) isotherms at 77 K on AC-90S (1), AC-90L (2), and AC-60L (3). Symbols show the experimental data; solid lines are the results of approximation.

Figure 3 shows the Type I isotherms of 77 K nitrogen adsorption in the synthesized AC samples, which are typical to microporous adsorbents [54]. All isotherms have a narrow hysteresis loop, which is indicative of weakly developed transport mesopores. As seen in Figure 3, the AC-90S with the highest burn-off degree displays the largest micropore volume, the value of which is proportional to the amount of nitrogen adsorbed at P/P_0_ = 1.

**Figure 4:**
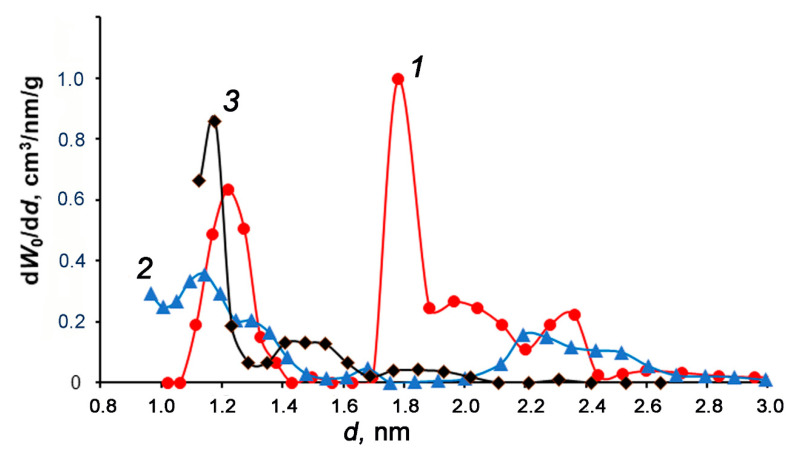
The pore size distribution of AC-90S (1), AC-90L (2), and AC-60L (3) calculated by the non-local density functional theory (NLDFT) model for a combined slit + cylinder pore geometry from the nitrogen adsorption data at 77 K. Symbols correspond to experimental data; solid lines are spline-approximation.

Figure 4 shows two pronounced maxima for the micropore width (diameter) of 1.25 and 1.8 nm for AC-90S. A wide micropore distribution within the range from 0.95 to 1.4 nm with a weak maximum at 0.95 and 1.15 nm is observed for AC-90L. In contrast, a narrow pore size distribution with one pronounced peak at 1.15 nm describes the porous structure of AC-60L. The NLDFT model data matched qualitatively the parameters evaluated by the D–R equation, which are summarized in Table 1.

The analysis of the AC textural properties with consideration for the activation conditions (see Figure 3 and Figure 4 and Table 1) revealed that the development of microporosity via the increase in the micropore volume from 0.44 to 0.64 cm^3^/g and BET surface from 1020 to 1470 m^2^/g, and the formation of transport mesoporosity up to 0.02 cm^3^/g should be attributed to the increased burn-off degree from 48 to 65 wt%. Thus, the results point to a significant effect of the char granule sizes and steam activation time on the formation of porosity in ACs through a widening mechanism: from narrow microporosity to a bimodal pore size distribution, including mesopores.

### 3.2. Morphology and Chemical Composition of CNS-Derived Carbon Adsorbents

**Figure 5:**
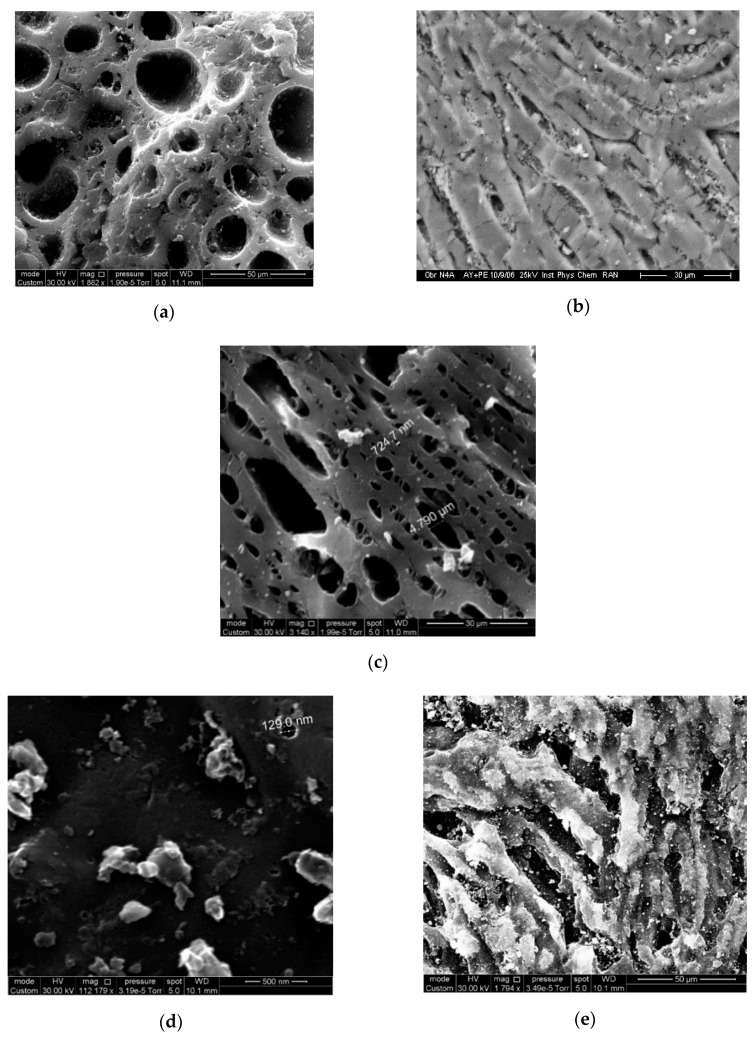
Scanning electron microscopy (SEM) images of transverse (**a**,**c**,**d**) and longitudinal (**b**,**e**) sections of the CNS-derived carbons with the different burn-off degree: AC-90S (**a**,**b**); AC-90L (**c**), and AC-60L (**d**,**e**). Note the difference in scale bars.

Figure 5a–e present the SEM images of transverse surface section (a, c, d) for AC-90S, AC-90L, and AC-60L and longitudinal surface section (b,d) for AC-90S, AC-60L, which are typical for ACs prepared from coconut shell [55,56]. The SEM images revealed an influence of the activation conditions on morphological features of carbon adsorbents. The changes in the original macromolecular network structure of coconut shell by its disruption and reconstruction into a new matrix structure were previously described in [56]. The increase in the burn-off degree leads to pitting and forming various channels, more pronounced in AC-90S and -90L. The shape of the channels is close to a cylinder (AC-90S, Figure 5a) or partially flattened cylinder (AC-90L, Figure 5c). The size of macropores (>50 nm) promotes the transport of adsorbate into the interior of AC granules. The transverse section of the rough surface of the AC-60L sample with the lowest burn-off degree contains small inclusions and pores of about 0.13 μm (Figure 5d). The longitudinal section of AC-60L (Figure 5e) shows an uneven surface with cracks similar to that of dried raw coconut shells [55,56].

Table 2 summarizes the data on the elemental composition of the AC samples. One can see that in addition to the dominating carbon, the CNS-derived adsorbents contain small amounts of oxygen (≤7 at%) and potassium (≤2 at%). The most activated AC-90S sample contains the highest amount of non-carbon impurity–potassium inherited from the precursor. As follows from [57], coconut shells contain about 1.2% K_2_O. Potassium contributes to surface alkalinity.

**Figure 6:**
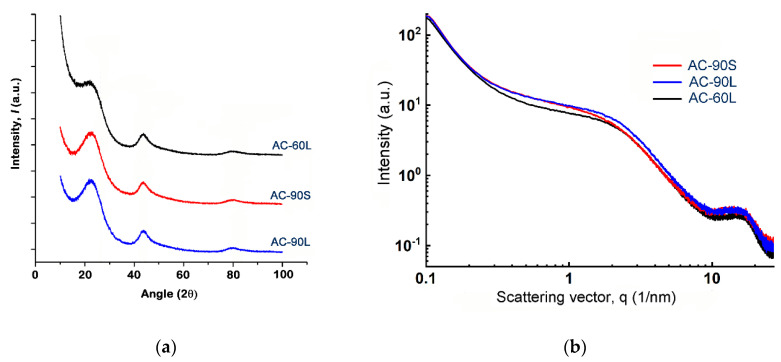
X-ray diffraction (XRD) (**a**) and small-angle X-ray scattering (SAXS) (**b**) patterns for the CNS-derived activated carbons.

Figure 6a,b show the XRD and SAXS patterns for the carbon adsorbents under study. According to XRD data (Figure 6a), the phase composition of all samples can be described as relatively large (~1–2 nm) graphite-like crystallites manifested by the broad Bragg peaks somewhat shifted from the positions inherent for graphite, which are embedded into an amorphous matrix. It is known that in the case of nanostructured sp^2^ carbons, evaluation of interlayer spacings and crystallite sizes from the position and width of Bragg reflections gives ambiguous results, and only approximate sizes can be obtained [58,59]. The resemblance of diffraction patterns for AC-90S to that for AC-90L with close burn-off degrees is indicative of their similar structural features. The AC-60L sample comprises a higher fraction of the amorphous matter, as suggested by a less prominent (002) peak. The micropore volume of AC-60L calculated from the N_2_ adsorption data (Table 1) is smaller than that of other samples. Thus, for this sample, a set of variations of XRD patterns as a function of the burn-off reflects the loss of volatile amorphous matter and ordering of the crystalline phase during the activation process. Figure 6b shows the SAXS intensity curves of all the samples, which are typical for activated carbons exhibiting a hierarchical system with micro, meso, and macropores e.g., [60]. The low-q region of the SAXS curve corresponds to scattering from relatively large crystallites, whereas a signal in the high-q range is caused by scattering from pores. Calculation of pore sizes using the model-independent Guinier radius [61] gave results close to that calculated on the assumption of a slit-like or cylindrical (rod-like) pore geometry: 0.6–0.65 nm. The relative fraction of pores is the smallest in the AC-60L sample (the plateau or intermediate region is the least intense) compared to AC-90L and AC-90S, which are similar. For the AC-90S sample, the plateau is less pronounced, which is indicative of dissimilarity of the pore size distribution from that in AC-90L. Thus, the variations of XRD patterns with the burn-off degree reflect the loss of volatile amorphous matter and ordering in the crystalline phase during the activation process.

### 3.3. Methane Adsorption on the CNS-Derived Carbon Adsorbents

**Figure 7:**
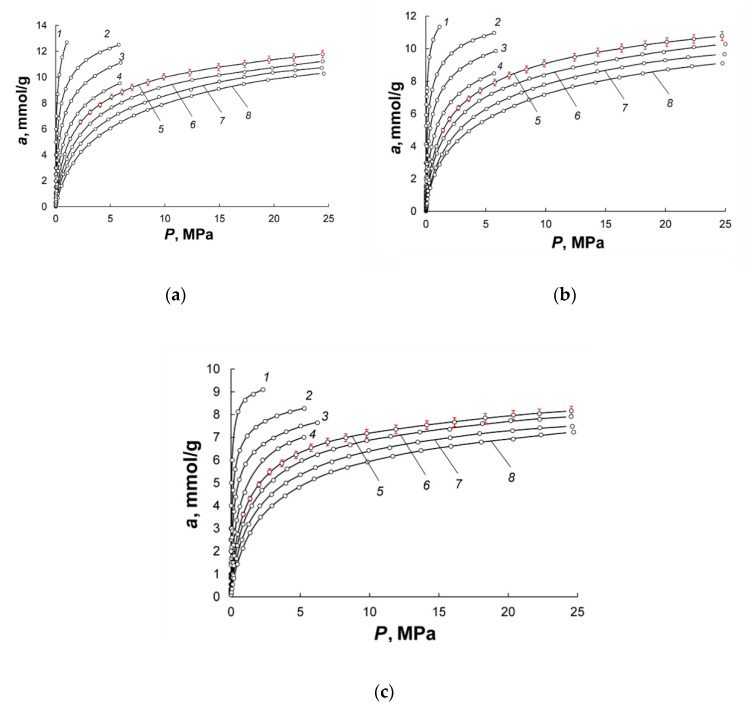
Methane uptake on AC-90S (**a**), AC-90L (**b**) and AC-60L (**c**) versus pressure at the temperatures, K: 178 (1); 216 (2); 243 (3); 273.15 (4); 300 (5); 320 (6); 340(7); 360 (8). Symbols show the experimental data; solid lines are the results of approximation by Equation (2). Error bars of 1.5% (5 Pa to 0.1 MPa), 3% (0.1–6 MPa) and 5% (6–25 MPa) are shown by red color.

Figure 7a–c show the isotherms of methane adsorption on the synthesized carbon adsorbents within the temperature range from 178 to 360 K and at pressures up to 25 MPa. The methane adsorption is reversible and increases with the volume of micropores.

The experimental data were approximated using the Bakaev equation [62] derived for the absolute adsorption isotherm:

(2) $$a\left(P\right)=\frac{k_{0\;}\left(k_{1}P+2k_{2}P^{2}+3k_{3}P^{3}\right)}{1+k_{1}P+k_{2}P^{2}+k_{3}P^{3}}.$$

Here, *k*_0_ characterizes the adsorption system, *k*_1_, *k*_2_, *k*_3_ are the temperature-dependent and numerically adjusted coefficients, P is the equilibrium pressure expressed in Pa. The maximum error of the regression of less than 3% allowed us to calculate a set of thermodynamic parameters with high accuracy. A comparison between the plots in Figure 7a–c shows that the AC-90S adsorbent with the highest burn-off degree and micropore volume displayed the most considerable methane uptake: at 300 K and 10 MPa, its methane adsorption capacity of about 10 mmol/g exceeds that of AC-90L and AC-60S: 9 and 7 mmol/g, respectively. Thus, at high pressures, AC-90S is the most effective adsorbent for methane. It should also be noted that the two-step steam activation produced the adsorbents with sufficiently high methane adsorption capacity compared to that of the carbon adsorbent prepared by thermochemical activation combined with steam activation [21].

**Figure 8:**
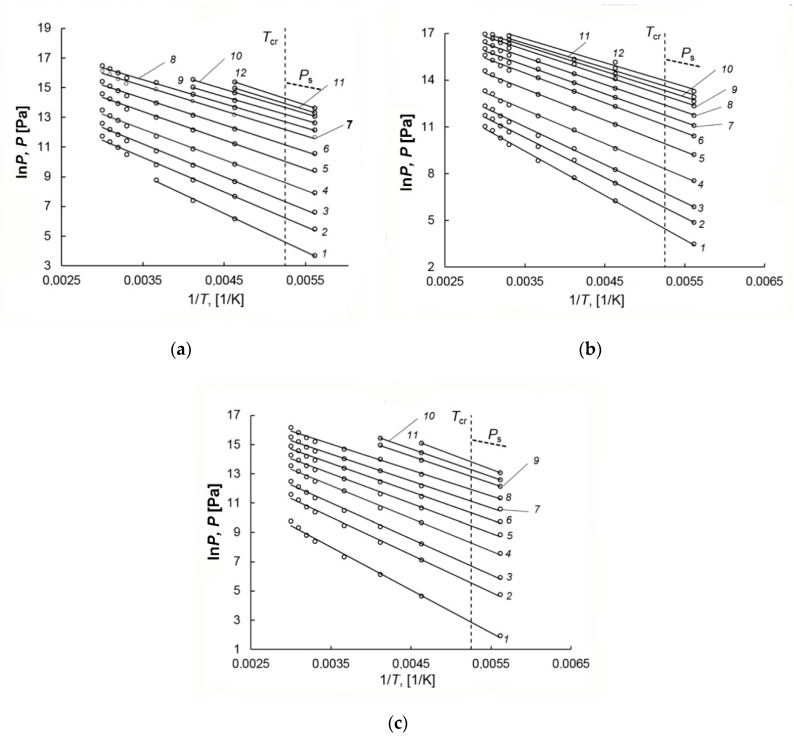
The isosteres of methane adsorption on: AC-90S (**a**) for the adsorption value *a* (mmol/g) of 0.2 (1), 0.5 (2), 1.0 (3), 2.0 (4), 4.0 (5), 6.0 (6), 8.0 (7), 9.0 (8), 10.0(9), 11.0(10), 11.5 (11), 12.2 (12); AC-90L (**b**) for *a* (mmol/g) of 0.3 (1), 0.6 (2), 1.0 (3), 2.0 (4), 4.0 (5), 6.0 (6), 7.0 (7), 8.0 (8), 9.0 (9), 9.5 (10), 10.0 (11), 10.5 (12); AC-60L (**c**) for *a* (mmol/g) of 0.1 (1), 0.5 (2), 1.0 (3), 2.0 (4), 3.0 (5), 4.0 (6), 5.0 (7), 6.0 (8), 7.0 (9), 7.5 (10), 8.0 (11).

The experimental methane adsorption isotherms were used to plot the isosteres, which are a quantitative relation between pressure and temperature at a constant value of adsorption (see Figure 8a–c). As follows from Figure 8a–c, the isosteres of methane adsorption on AC-90S, A-90L, and AC-60L plotted as lnP versus 1/T are well approximated by a straight line. The linearity of the isosteres holds when the temperature passes through the critical point of methane *T*_cr_ = 190.77 K to the region of the non-ideality of a gaseous phase. This fact is a clear indication of a peculiar state of highly dispersed methane in micropores, which differs significantly from the non-ideal gaseous phase [50,63]. In this case, the number of methane molecules does not exceed 10–20 in a micropore with a strong adsorption field created by the opposite walls, which excludes the formation of a liquid phase. Many researchers, for example, Bering et al. [64,65,66], Barrer et al. [67], Fomkin [68,69], Bülow et al. [70] observed linear adsorption isosteres for numerous vapors and gases, including methane [40,41,42,71] and inert gases [72] adsorbed in microporous adsorbents. It was reported in [73] that the property of the linearity of adsorption isosteres in microporous adsorbents could be extended to the range of the compressed liquid state. Therefore, the highly dispersed state of adsorbed methane is responsible for methane storage in micropores without phase transition over wide intervals of super- and subcritical temperatures and pressures. The linear isosteres were used to calculate thermodynamic parameters of methane adsorption in the adsorbents, namely, differential molar isosteric heats of adsorption.

### 3.4. Differential Molar Isosteric Heat of Methane Adsorption on the CNS-Derived Carbon Adsorbents

As follows from its definition [69,74], the isosteric heat of adsorption, *q*_st_, is a difference between the molar enthalpy of the equilibrium gas phase *h*_g_ and differential molar isosteric enthalpy of the adsorption system *H*_a_:

*q*_st_ = *h*_g_ − *H_a_*. (3)

The studies concerning the evaluation of thermodynamic functions of adsorption [41,71,72,75,76] provided evidence that Equation (4) by Bakaev [77], which accounts for the factors affecting the value of differential molar isosteric heat of adsorption, enables one to calculate its value correctly from adsorption data:

(4) $$q_{st}\;=\;-R\cdot Z\cdot {\left[\frac{\partial \left(lnP\right)}{\partial \left(1/T\right)}\right]}_{a}\cdot \left[1-{\left(\frac{\partial v_{a}}{\partial a}\right)}_{T}{/}_{g}\right]-{\left(\frac{\partial P}{\partial a}\right)}_{T}\cdot \left[v_{a}-T\cdot {\left(\frac{\partial v_{a}}{\partial T}\right)}_{a}\right].$$

Here, *Z* = *P*⋅*ν*_g_/(*RT*) is the coefficient of compressibility of the equilibrium gas phase at pressure *P* (Pa) and temperature *T* (K); *ν*_g_ is the specific gas phase volume, m^3^/kg; *R* is the universal gas constant, J/(mol·K); *v*_a_ = *V*_0_(*P,T*)/*m*_0_ is the reduced volume of the adsorbent–adsorbate system, cm^3^/g; and *V*_0_ and *m*_0_ are the volume and mass of the regenerated adsorbent, respectively. Thus, the Bakaev Equation (4) includes all the effects caused by non-ideality of a gaseous phase and non-inertness of an adsorbent: adsorption isothermal deformation (*∂v_a_*/*∂a*)*_T_*, temperature isosteric deformation (*∂v_a_*/*∂T*)*_a_*, the slopes of the adsorption isotherm (*∂P*/*∂a*)*_T_* and isostere [*∂lnP*/*∂*(1/*T*)]*_a_*, and non-ideality of a gas phase *Z* [77].

The estimations for ACs with close textural parameters revealed that at supercritical temperatures, the corrections for adsorption-induced deformation [75,76] and temperature deformation [78] of adsorbent could be ignored in calculating *q*_st_. Therefore, the Bakaev equation is reduced to a formula, which takes into account the non-ideality of the gaseous phase and steepness of adsorption isotherm:

(5) $$q_{st}\;=\;-R\cdot Z\cdot {\left[\frac{\partial \left(lnP\right)}{\partial \left(1/T\right)}\right]}_{a}-{\left(\frac{\partial P}{\partial a}\right)}_{T}\cdot v_{a}.$$

When calculating the differential molar isosteric heat of adsorption, its initial value, *q*_st_^0^, was evaluated using Equation (2) at *P* → 0:

(6) $$a{\left(P\right)}_{P\to 0}=k_{0}k_{1}P=K_{\Gamma }P.$$

As follows from [76], the initial isosteric heat of adsorption can be found from the temperature dependence of the Henry constant *K*_Γ_:

(7) $$q_{st}^{0}\;=\;R\left[\frac{\partial \left(lnK_{I}\right)}{\partial (1/T)}\right]$$

At the initial stage of adsorption, the isosteric heat of methane adsorption on AC-90S, AC-90L, and AC-60L is independent of temperature and amounts to 23.2, 24.7, and 24.7 kJ/mol, respectively.

**Figure 9:**
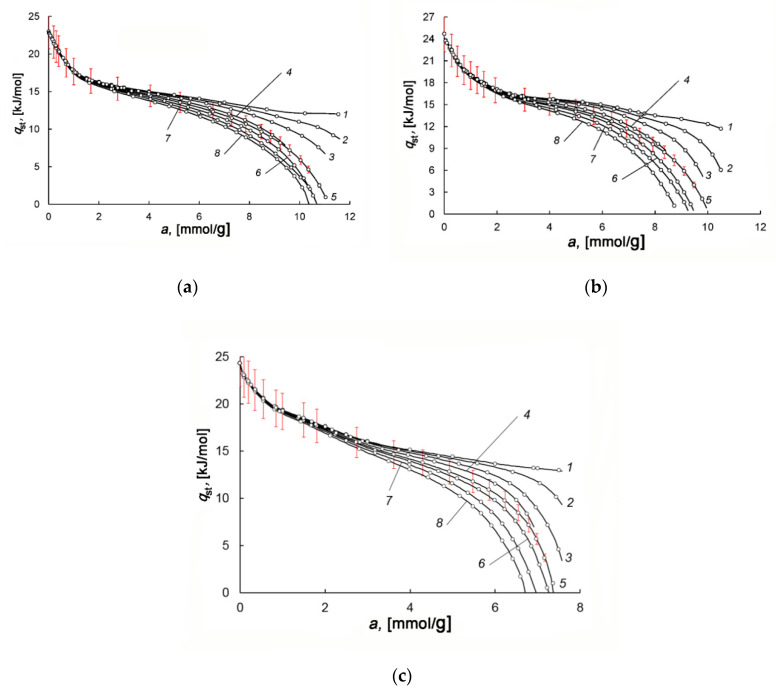
The differential molar isosteric heat of adsorption versus the value of methane adsorption in AC-90S (**a**), AC-90L (**b**), and AC-60L (**c**) at temperatures, K: 178 (1), 216 (2), 243 (3), 273.15 (4), 300 (5), 320 (6), 340 (7), 360 (8). Symbols show experimental data; solid curves are the results of approximation by Equation (5). The error bar is 10%.

Figure 9a–c demonstrate the dependences of the isosteric heat of methane adsorption on AC-90S, -90L, and -60L within a wide temperature range. The behaviors of *q*_st_ = *f*(*a*) are the same for all the adsorbents, i.e., almost independent of their specific micropore volume, packing density, and chemical composition. At the early stage of adsorption, the curves *q*_st_(*a*) for different temperatures coincide, but they diverge with an increase in micropore loading. A resulting “fan” of the curves is caused by a temperature-dependent contribution from the coefficient of compressibility of the equilibrium gas phase in Equation (5). Since the adsorption systems are studied within almost the same *P,T*-intervals, the difference in the absolute values of *q*_st_ is related to the various values of methane adsorption.

It should be noted that the behaviors of *q*_st_ = *f*(*a*) are typical to gas adsorption in microporous adsorbents with high-energy adsorption sites. The similar dependences *q*_st_ = *f*(*a*) were observed for adsorption of carbon dioxide and methane in Na-ZSM-5 and NaX zeolites [79], carbon dioxide in silicalite [80], methane in microporous-activated carbons [69,73,81], and even for adsorption of inert gases as neon [82] in rutile and krypton in AC [83]. For all these adsorption systems, the high values of *q*_st_ are observed at the early stages of adsorption, when gas molecules occupy a large portion of micropores by binding to high-energy adsorption sites. Thus, at low adsorbate loadings, the absolute value of *q*_st_ depends on the density of adsorption sites, which are high-energy micropores with dimensions comparable to that of adsorbate molecules, and the surface heteroatoms, including metal ions. It should be noted that metal ions are associated with electrostatic interactions, which are likely to be of minor importance for methane, having no dipole or quadrupole moment. With the increase in the number of adsorbed molecules, the high-energy adsorption sites are completely occupied, and the adsorbate–adsorbate interactions contribute to the heat of adsorption. Thus, the dependence *q*_st_ = *f*(*a*) reflects the transformation in the state of adsorbed molecules upon the adsorption process from binding with the high-energy adsorption sites to the formation of molecular associates and their subsequent rearrangement close to saturation.

### 3.5. Thermodynamic Characteristics of the Adsorption Storage System

Thermodynamic parameters of the ANG system, notably the enthalpy, determine its thermal behaviors, including heating-up under various operational conditions. Here, the total enthalpy is a sum of enthalpies of individual elements: a methane/AC adsorption system, gaseous phase, metal tank, and heat exchangers, thermal insulation (we neglected it in the calculations), etc. The enthalpy of the methane/AC system *H_a_* is calculated by integrating Equation (3), which includes the differential enthalpy of the adsorption system. The enthalpy as a state function depends only on the final and initial value and not on the path taken to reach the final value. Therefore, we choose the straightforward path of Equation (3) integration–isothermal adsorption from zero loadings of a regenerated adsorbent to a final value of adsorption. Since only the changes in the enthalpy are relevant, its reference value can be chosen arbitrarily and separately for each element of the ANG system. Thus, the enthalpy of the adsorbent–adsorbate system *H_a_* for isothermal equilibrium adsorption is calculated as follows [38,39]:

(8) $$H_{a}=\;\left[c_{c}\cdot \left(1-x\right)\cdot \left(T-T_{0}\right)+c_{b}\cdot x\cdot \left(T-T_{0}\right)+\left(1-x\right)\cdot \int_{0}^{a}\left(h_{g}-q_{\mathrm{st}}\right)d\left(a\right)\right]\cdot \rho_{p}\cdot V_{\tan k},$$

where *c*_c_ and *c*_b_ are the specific heat capacities of regenerated carbon adsorbent without methane and a polymer binder, respectively; *x* is the mass content of a binder in the monolithic adsorbent; *T* is the temperature of the adsorption system; *T*_0_ is the arbitrary reference temperature, for example, 273.15 K; *a* is the adsorption value reduced to a “pure” adsorbent without the binder, i.e., it corresponds to the plot in Figure 7; *ρ*_p_ is the packing density of adsorbent on the volume of the system; *V*_tank_ is the internal volume of the ANG tank.

The enthalpy of the total ANG system for isothermal equilibrium adsorption is expressed by a sum:

(9) $$H_{\mathrm{ANG}}=\;H_{a}+H_{g}+H_{\tan k}=\;H_{a}+h_{g}\cdot \varepsilon \cdot V_{\tan k}+c_{\tan k}\cdot m_{\tan k}\cdot \left(T-T_{0}\right),$$

where *ε* is the porosity, i.e., fractional void space of the tank; *c*_tank_ is the average specific heat capacity of the tank elements (housing plus heat exchangers); for simplicity, it was taken as a constant.

### 3.6. Mathematical Model of the Gas Charge/Discharge Processes in the ANG System

In this work, we considered a relatively simple model with lumped parameters, which did not take into account the thermal and diffusional gradients in an adsorbent bed, and between the adsorbent and the adsorber walls. These simplifications were justified because we considered slow charge/discharge processes, unlike a fast circuit charge process of the ANG system described by a more complicated model developed in the recent study [33].

The model is based on the mass and energy balance equations:

(10) $$∆m_{g.\mathrm{acc}}=\;∆m_{g.\mathrm{in}}+∆m_{g.\mathrm{out}},$$

(11) $$∆H_{\mathrm{acc}}=\;∆H_{g.\mathrm{in}}+∆H_{g.\mathrm{out}}+∆Q_{\mathrm{he}},$$

where Δ*m*_g.acc_ and Δ*H*_acc_ are the changes in the mass of stored gas in the ANG system and the enthalpy of the ANG system, respectively; Δ*m*_g.in_ and Δ*m*_g.out_ are the masses of incoming and outgoing gas, respectively; Δ*H*_g.in_ and Δ*H*_g.out_ are the enthalpies of incoming and outgoing gas, respectively; Δ*Q*_he_ is the amount of heat transferred upon the exchange with the surroundings and coolant in the heat exchangers.

Equations (10) and (11) were solved using successive approximations at every time step. Assuming constant pressure of supplied or delivered gas after the reducer, we did not include technical work in Equation (11). The flow rates of supplied and delivered gas were assumed to be constant during initial periods of filling and emptying the tank until the pressure reaches the maximum and minimum values. This assumption is close to realistic experimental conditions when gas supply and delivery rates are determined by a compressor capacity.

The amount of heat transferred upon heat exchange in the period Δτ is given by the following equation:

(12) $$∆Q_{\mathrm{he}}=\;k_{e}\cdot F_{e}\cdot \left(T_{f}-T\right)\cdot ∆\tau =\;K_{e}\cdot \left(T_{f}-T\right)\cdot ∆\tau ,$$

where *k_e_* is the effective heat transfer coefficient related to the heat transfer surface area *F_e_* and the difference between the ambient or coolant temperature *T_f_* and average adsorber temperature *T*.

The coefficient $K_{e}=k_{e}\cdot F_{e}$, [W/K] characterizes the intensity of cooling or heating of the total ANG system. The coefficients *k_e_* and *K_e_* are assumed to be constant, although their magnitudes depend on many parameters, primarily on the pressure in the adsorber. This simplification is permissible since the pressure (and the gas density) in the adsorber changes significantly only in the short initial periods of the charge/discharge cycle.

With the forced coolant circulation, almost the entire external heat gain is compensated by the coolant. Therefore, no additional factor related to the heat exchange with the surroundings should be involved in Equation (12).

The mass of gas stored in the adsorber is calculated by a formula:

(13) $$m_{acc}=\;a\cdot \rho_{p}\cdot \left(1-x\right)\cdot V_{\tan k}+\varepsilon \cdot \rho_{g},$$

where *ρ*_g_ is the gas density at the average temperature and pressure of the adsorber.

The amount of gas stored in the ANG system in standard volume units of m^3^(STP) is calculated as follows:

(14) $$V_{\mathrm{acc}}=\;m_{\mathrm{acc}}/\rho_{\mathrm{STP}},$$

where *ρ*_STP_ is the gas density at STP conditions determined by standard temperature (293.15 K) and pressure (101,325 Pa).

### 3.7. Experimental Study of Energy and Capacity Parameters of the ANG System

Following the method described in Section 2.4, the investigations of the charge/discharge processes were carried out for the ANG system loaded with the microporous AC-90S adsorbent under two different conditions:

1. thermal insulation regime realized by thermal insulation and in the absence of forced heating and cooling. The initial period of charge and discharge proceeds under nearly adiabatic conditions;
2. hermal regulation regime realized by forced cooling and heating using a coolant with a temperature close to the ambient.

Experimental data were analyzed using a developed mathematical model (see Section 3.5). This model allowed us to compare the intensities of cooling and heating under the charge/discharge conditions and calculate the amounts of accumulated gas.

#### 3.7.1. The Charge/Discharge Processes under the Thermal Insulation Conditions: Model and Experimental Data

**Figure 10:**
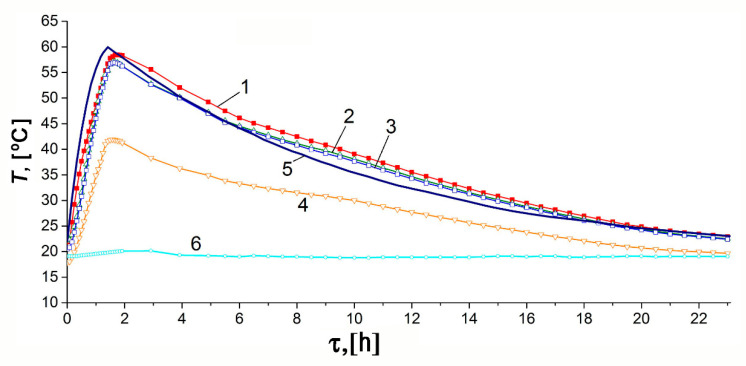
Temporal variations of the temperature inside (TE1) and outside the adsorber during the charge cycle under the thermally insulated conditions: curves 1–4 correspond to the TE1–4 sensors readings, respectively; curve 5 is the average adsorber temperature calculated from the model, curve 6 is the ambient temperature.

**Figure 11:**
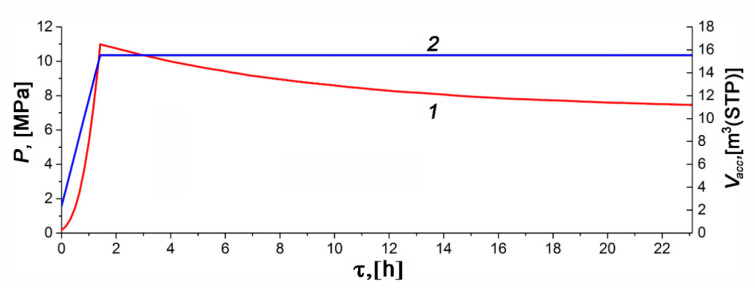
Temporal variations of pressure (1) and the amount of gas stored in the ANG tank (2) during the charge cycle calculated for the thermally insulated conditions.

Figure 10 and Figure 11 show the temporal variations of temperature and pressure in the ANG system during the thermally insulated charge process, respectively. In this case, the thermally insulated tank is cooled only due to low-intensity heat exchange with the surroundings. The charging procedure was carried out in steps. In the beginning, the gas was loaded up to 10–11 MPa for about 1.5 h, followed by extended cooling without gas supply. Final refueling up to 10 MPa was executed on the next step. Figure 10 and Figure 11 demonstrate the plots related to the first step of the charging process.

The results of mathematical modeling are in agreement with the readings of most sensors. The sensor TE1 located inside the adsorber (see Figure 2) most accurately describes its internal heat state. At least, this is true in the absence of coolant flow through the adjacent heat-exchanger. A small difference between the readings of the TE2 sensor and the computed average temperature of the adsorber can be attributed to the uncertainty of the model, and intensive heat exchange during the charge cycle due to natural convection inside the adsorber. Significant inertia both of the temperature sensors and the internal heat-exchanging processes, which make the temperature field uniform, is responsible for the deviation of a “peak” time from the end of the charging cycle embedded in the mathematical model. The readings of the TE4 sensor located at the bottom (see Figure 2) show the most considerable deviation from the model and readings of other sensors. These deviations are due to the design features of the lower part of the adsorber responsible for high thermal capacity and extended external heat exchange surface. Other explanations can also be offered: convection (the adsorber is vertical, see Figure 2), a great distance between the sensor and gas inlet located in the upper part, etc. The discrepancy between the shape of the experimental and model curves can be explained by the model simplifications and a more complex heat exchange process inside the adsorber.

The temperature inside the adsorber increases from 21.9 to 58.5 °C during the charge cycle. About 21 h after the start of the charge cycle, the TE1 sensor showed that the temperature inside the adsorber differed by 5 °C from the ambient temperature (asymptotic temperature). We used this result as a criterium of the charge termination time (first step of the charge cycle) when comparing various regimes of heat transfer.

The model calculations were carried out for the effective heat transfer coefficients *K_e_* = 4 W/K or *k_e_* = 2.85 W/m^2^·K relative to the inner surface of the adsorber of 1.4 m^2^ or *k_e_* = 2.22 W/m^2^ relative to the outer surface of the tank of 1.8 m^2^. We expected the heat transfer coefficient of about 1.8–2.3 W/m^2^·K for the outer surface, which corresponded to the results of the mathematical modeling.

**Figure 12:**
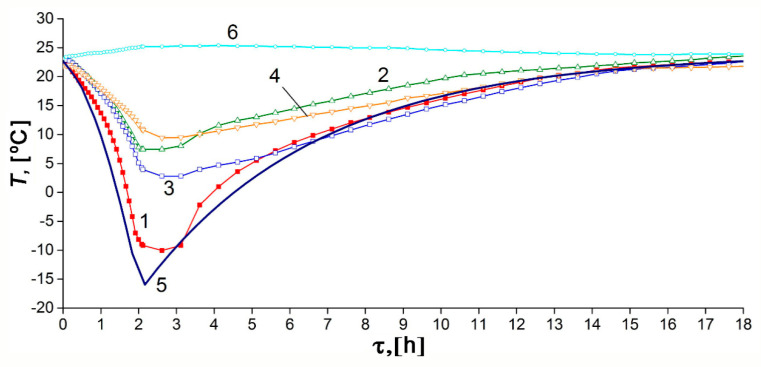
Temporal variations of the temperature inside and outside the adsorber during the discharge cycle under the thermally insulated conditions: curves 1–4 correspond to the TE1–4 sensors readings, respectively; curve 5 is the average adsorber temperature calculated from the model, curve 6 is the ambient temperature.

Figure 12 shows the variations of the experimental temperature readings and the model average temperature with the discharge time under the thermal insulation conditions: low-intensity heat exchange with the surroundings and absence of coolant flow. The discharge cycle was also performed in steps: at the first step shown in Figure 12, the pressure drop to 0.19 MPa was followed by slow heating, maintaining the amount of gas. During the discharge cycle, the temperature inside the adsorber decreased from 22.7 °C to −10.1 °C. After a period of discharge of 12 h 10 min, it differed by 5 °C from the ambient temperature, which can be considered as a conditional end of the discharge cycle (the first step).

The temperature gradients, arising during discharge along the radius of the adsorber (between TE1 and other sensors) and its length or height (between TE2, TE3, and TE4), are more noticeable than in the charge cycle. This fact is attributed to the lower average pressure, which reduces convection inside the tank. This is probably the reason for the discrepancy between the experimental data and model: temperature gradients can be complex, and the adsorbent bed may have a temperature lower than the reading of TE1 and TE3. The impact of impurities in natural gas can also be more pronounced during discharge. The temperature drop in the central part of the adsorber was most significant (TE1 and TE3), probably due to better thermal insulation. The lower part of the adsorber (TE4) is noticeably colder than the upper part (TE2) in the final stage of cooling, which is a consequence of convection.

**Figure 13:**
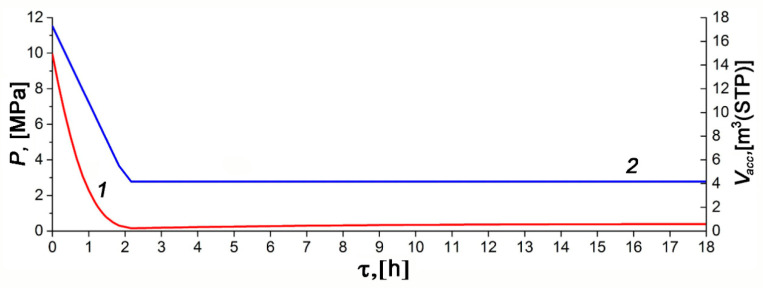
Temporal variations of pressure (1) and the amount of gas accumulated in the adsorber (2) during the discharge cycle calculated for the thermally insulated conditions.

Results for the discharge process were calculated with the effective heat transfer coefficients *K_e_* = 6.3 W/K or *k_e_* = 4.5 W/m^2^·K relative to the inner surface of the adsorber (1.4 m^2^) or *k_e_* = 3.5 W/m^2^ relative to the outer surface of the tank (1.8 m^2^). As follows from the mathematical modeling (Figure 13), at the first stage of discharge, the ANG system delivers only 75% of the accumulated gas. The same result is expected in practice in the absence of specialized heating facilities.

#### 3.7.2. The Charge/Discharge of the ANG System under the Thermal Regulation Conditions: Model and Experimental Data

**Figure 14:**
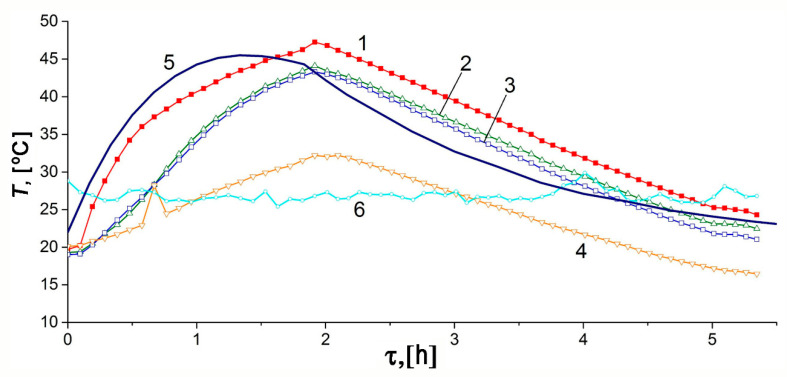
Temporal variations of the temperature inside and outside the ANG tank during the thermally regulated charge cycle: curves 1–4 correspond to the TE1–4 sensor readings, respectively; curve 5 is the average adsorber temperature calculated from the model, curve 6 is the ambient temperature. The average coolant temperature is 20 °C.

The thermally regulated charge/discharge processes imply the use of the internal and external heat exchangers (see Figure 1 and Figure 2). Figure 14 demonstrates the experimental and calculated time variations of adsorber temperatures during a thermally regulated charge cycle with the forced circulation of the coolant with a temperature of 20 °C. The charge cycle was carried out in steps, the first of which is shown in Figure 14. The temperature gradients in the radial direction are larger than that in the thermally insulated charge cycle. A plausible suggestion is a difference in the performance of the internal and external heat exchangers. One should note the significant discrepancy of the TE4 readings from the other sensors and a drop of its temperature below the target value of 20 °C. Several possibilities can explain these effects; first, the sensor inaccuracy, second, non-uniform cooling along a vertical axis of the adsorber by the external heat exchanger; third, convection flows inside the adsorber when the gas layers cooled by the internal heat exchanger move to the bottom of the adsorber.

**Figure 15:**
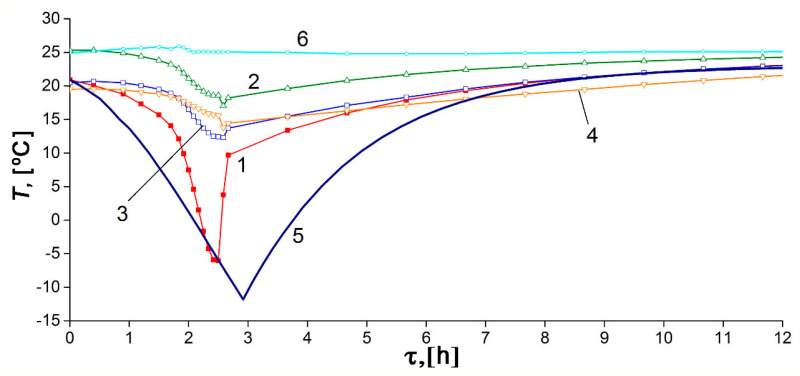
Temporal variations of the temperature inside and outside the adsorber during the thermally regulated discharge cycle: curves 1–4 correspond to the TE1–4 sensors readings, respectively; curve 5 is the average adsorber temperature calculated from the model, curve 6 is the ambient temperature. The average coolant temperature is 23 °C.

In general, the mathematical model adequately describes this regime. Figure 15 demonstrates significant inertia in the sensor readings caused by the temperature gradients that really exist in the system. At the same time, the absolute values of heating of the adsorber correspond to realistic sensor readings. The results of the mathematical model were obtained with the effective heat transfer coefficients *K_e_* = 20 W/K or *k_e_* = 12.7 W/m^2^·K relative to the gas heat exchange surface (1.57 m^2^), i.e., the sum of the external surface of the internal heat exchanger and the internal surface of the cooled adsorber; or *k_e_* = 19.2 W/m^2^·K relative to the heat exchange surface for the coolant, i.e., the total internal surface of both heat-exchangers of 1.04 m^2^. These values were averaged since it was evident that the performance per unit area of the internal heat-exchanger was higher than that of the external heat-exchanger.

During the charge cycle, the temperature inside the adsorber increased from 19.7 to 47.3 °C. After 5 h 10 min, the temperature inside the adsorber was 25 °C which was 5 °C higher than the average coolant temperature. By this time, the readings of other sensors were noticeably lower than this value. Therefore, the average adsorber temperature could already reach 25 °C before the TE1 sensor reacted. However, it was impossible to establish this fact with certainty in this experiment. Obviously, the thermally-regulated charge with forced cooling appeared to be more efficient than the thermally insulated charge because the charge time, including the cooling period, was reduced by 4 times. Nevertheless, the charge time of 5 h 10 min is too long, which is not suitable for many practical applications. It can be shortened by increasing the heat exchange surface or creating internal channels in the monolithic adsorbent.

Figure 15 shows the temperature variations during the discharge cycle with forced heating by the coolant flow with an average temperature of 23 °C. In this case, the temperature gradients were strongly pronounced and were caused by low pressure in the system (weak convection) and the significant impacts of the heat-exchangers on the sensor readings. The mathematical model was in good agreement with the experimental variations of adsorber temperature. The effective heat transfer coefficients *K_e_* = 14 W/K or *k_e_* = 8.9 W/m^2^·K relative to the gas heat exchange surface or *k_e_* = 13.5 W/m^2^·K relative to the heat exchange surface for coolant were used.

With the forced heating, the adsorber temperature changed less significantly than at the thermally insulated conditions: the peak TE1 values drops to −6 from initial 20.9 °C. Therefore, the total temperature drop was 26.9 °C, whereas under the thermal insulation conditions: it was 32.8 °C. After 6 h 40 min, the deviation between the temperature readings of all sensors and 23 °C (the coolant temperature) was less than 5 °C. This moment can be considered as a conditional end of thermal regulation during the discharge cycle (the first stage). The process occurred two times faster than under the thermal insulation conditions.

Table 3 summarizes the directly measured amounts of methane delivered under the conditions of thermal insulation/regulation. They are smaller than the calculated values by 6/4%, respectively. The simplest explanation of these discrepancies is nitrogen impurity in natural gas (up to 2%), which affected the experimental data, whereas the model considered pure methane. The volumetric delivery capacity of the ANG system obtained in our experiments is about 150 m^3^(STP)/m^3^ at the pressure drop from 0.1 to 10 MPa.

## 4. Conclusions

Microporous activated carbons were produced by carbonization of coconut shells followed by steam activation of the granular char at 1123–1273 K. A comparative analysis of the data on nitrogen adsorption at 77 K by the D–R, BET, Kiselev equations, and NLDFT model was performed. It was shown that the activation time of 90 min and small granule sizes were sufficient for producing the AC-90S carbon adsorbent with a well-developed microporosity characterized by the high values of micropore volume of 0.62 cm^3^/g, BET surface of 1470 m^2^/g, and a low percentage of mesoporosity. The XRD patterns indicated that the increase in the burn-off to 66% led to some growth of graphite-like crystallites embedded in the amorphous matrix. The AC-90S adsorbent contains a high amount of non-carbon impurities, which affect its surface chemistry. The data on methane adsorption measured at the temperatures varied from 178 to 360 K and pressures up to 25 MPa confirm that the significant methane adsorption capacity of AC-90S is determined by the high values of micropore volume and standard characteristic energy of adsorption. Thus, the combination of textural properties explains the sufficiently high methane storage capacity of AC-90S. The simple synthesis procedure also makes AC-90S a good candidate for real ANG systems. The AC-90S was chosen as an adsorbent in the experimental test adsorber with a volume of 98 L developed for evaluating the thermodynamic parameters of the ANG system. The internal and external heat-exchangers, and the temperature sensors inside the adsorber filled with the compacted AC-90S adsorbent, allowed thorough examination of the temperature distribution over the adsorber during the natural gas charge/discharge cycles under two different heat exchange conditions: thermal insulation and thermal regulation. A simple mathematical model based on thermodynamic parameters of the methane/AC-90S adsorption system was used to simulate the charge/discharge processes in the ANG tank under the same conditions of heat exchange. The results of modeling are consistent with the experimental data. The experimental data combined with the mathematical model enabled evaluating the integral heat-exchange characteristics, which determined the duration and efficiency of charge/discharge processes. The forced thermal regulation made it possible to accelerate the charge/discharge processes by 2/4 times and to obtain slightly larger amounts of delivered methane compared to the thermal insulation process. The mathematical model and the evaluated heat exchange coefficients and temperature fields, can be directly used in elaborating the optimal heat-exchange facilities of the ANG system to maximize its performance.

## Figures and Tables

**Table 1:** The parameters of the porous structure of the coconut shell (CNS)-derived AC adsorbents with the various burn-off degree, packing density, and globular sizes evaluated from adsorption data by the Dubinin–Radushkevich (D–R), Brunauer–Emmet–Teller (BET), and Kelvin equations.

Sample	*W*_0,_ cm^3^/g	*x*_0_, nm	*E*_0_, kJ/mol	*S*_BET_, m^2^/g	*W*_S,_ cm^3^/g	*W*_meso,_ cm^3^/g	d, g/L	∆, mm	Ω, wt.%
AC-90S	0.64	0.58 */0.79 **	20.1 */15.1 **	1470	0.66	0.02	380	0.7−1.1	65
AC-90L	0.54	0.56 */0.64 **	21.6 */18.8 **	1270	0.55	0.01	465	0.9−2.4	60
AC-60L	0.44	0.59	20.3	1020	0.44	0.00	530	1.9−3.0	48

*–first mode; **–second mode.

**Table 2:** Elemental chemical composition of the CNS-derived activated carbons, at%.

Sample	C	O	K
AC-90S	92.0	6.0	2.0
AC-90L	93.0	6.5	0.5
AC-60L	96.0	3.5	0.5

**Table 3:** Experimental and modeling data on the amount of delivered natural gas during the discharge process under the conditions of thermal insulation and thermal regulation.

Parameters and Indicators of the Discharge Process	Discharge Conditions	
	Thermal Insulation	Thermal Regulation
Initial pressure, MPa	9.94	10.04
Initial adsorber temperature (TE1 reading), °C	22.7	20.9
Final pressure, MPa	0.1	0.1
Final temperature after the termination of the discharge cycle (TE1 reading), °C	24.2	24.5
Delivered natural gas (experimental), m^3^ (STP)	14.8	15.2
Delivered natural gas (model), m^3^(STP)	15.7	15.9
